# Influence of linearly polarized light on the transverse relaxation of ground-state ^133^Cs atoms

**DOI:** 10.1038/s41598-024-62853-y

**Published:** 2024-05-30

**Authors:** Zhichao Ding, Jie Yuan

**Affiliations:** 1 College of Ordnance Engineering, Naval University of Engineering, Wuhan, 430033 China; 2 College of Advanced Interdisciplinary Studies, National University of Defense Technology, Changsha, 410073 China

**Keywords:** Physics, Optical physics, Quantum physics

## Abstract

In order to obtain an understanding of the relationship between the optical absorption and the transverse relaxation, the influences of linearly polarized light respectively at ^133^Cs D1 and D2 lines on the transverse relaxation of ground-state ^133^Cs atoms are studied. Under different vapor temperatures, light intensities and light frequencies, transverse spin relaxation times are separately measured for ^133^Cs atoms in different hyperfine levels. For theoretically analyzing the measuring results, especially for an unusual trend that the transverse spin relaxation time rises with the increase of light intensity, photon absorption cross-sections of linearly polarized light by ^133^Cs atoms are simulated. The experimental results show that through influencing the optical absorption and spin-exchange collisions, the linearly polarized light plays a remarkable role in the transverse spin relaxation. The results obtained by this paper can provide a guide to find the optimal intensity and frequency of linearly polarized light in practical applications for decreasing the influences of linearly polarized light on the transverse relaxation.

## Introduction

Owning to the special nature of alkali atoms, they can be easily spin-polarized by optical pumping^1,2^. The spin-polarized alkali atoms can be widely found in many practical applications, ranging from quantum memory and teleportation to atomic magnetometers and atomic gyroscopes^3–7^. In these applications, for obtaining a good performance, a low transverse spin relaxation and a high spin polarization are desired in usual cases for alkali atoms^5–7^. However, some mechanisms such as spin-destruction collisions, spin-exchange collisions, and optical absorption, will lead to the transverse spin relaxation, and further limit the spin polarization which can be achieved by optical pumping^8–10^. Therefore, getting a better picture of the influences of some spin-relaxation mechanisms on the transverse spin relaxation is significant.

As for some spin-relaxation mechanisms of alkali atoms, their influences on the transverse spin relaxation have been widely discussed, and many practical methods are proposed to lower their influences^8–13^. For examples, in order to suppress the relaxation caused by radiation trapping and wall collisions, quenching gas and buffer gas are added to the alkali vapor cell, respectively^11,12^. In addition, through proper optical pumping, most alkali atoms can be regulated into a specific level, the transverse spin relaxation caused by spin-exchange collisions can be diminished to a great extent^13^. However, for the relaxation due to optical absorption, it is difficult to find an efficient approach to suppress it since a compromise between increasing the spin polarization or signal amplitude and decreasing the transverse spin-relaxation rate usually needs to be made^10,14,15^.

Optical absorption involves the processes of optical pumping and optical detection in the practical applications. In these processes, the alkali atoms in ground state will first be excited to the high levels, and then decay back to different ground-state levels quickly by quenching or spontaneous radiation, making the decoherence of atomic precession and thus leading to the transverse spin relaxation^2,8^. In addition, optical absorption will change the proportion of ground-state alkali atoms in two hyperfine levels, influencing the spin-exchange collisions, while the spin-exchange collisions are one main factor which cause the transverse spin relaxation, especially when the operating temperature is high^2,10,16^. So, the spin-exchange collisions influenced by optical absorption is another important way leading to the transverse spin relaxation, indicating the complexity of the relationship between the optical absorption and the transverse spin relaxation.

In order to realize optical pumping, circularly polarized light is used to prepare atomic spins, while in order to realize optical detection, circularly or linearly polarized light is adopted to monitor atomic spins^2^. In Ref.^17^, the ^133^Cs atom is selected, and the influences of circularly polarized light on the transverse spin relaxation of different ground-state ^133^Cs atoms are observed. In this paper, in order to obtain an understanding of the relationship between the optical absorption and the transverse relaxation, the influence of linearly polarized light on the transverse spin relaxation of different ground-state ^133^Cs atoms is investigated.

## Methods

**Figure 1:**
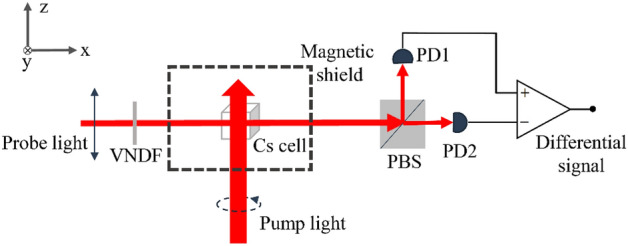
Schematic diagram of the experimental setup.

**Figure 2:**
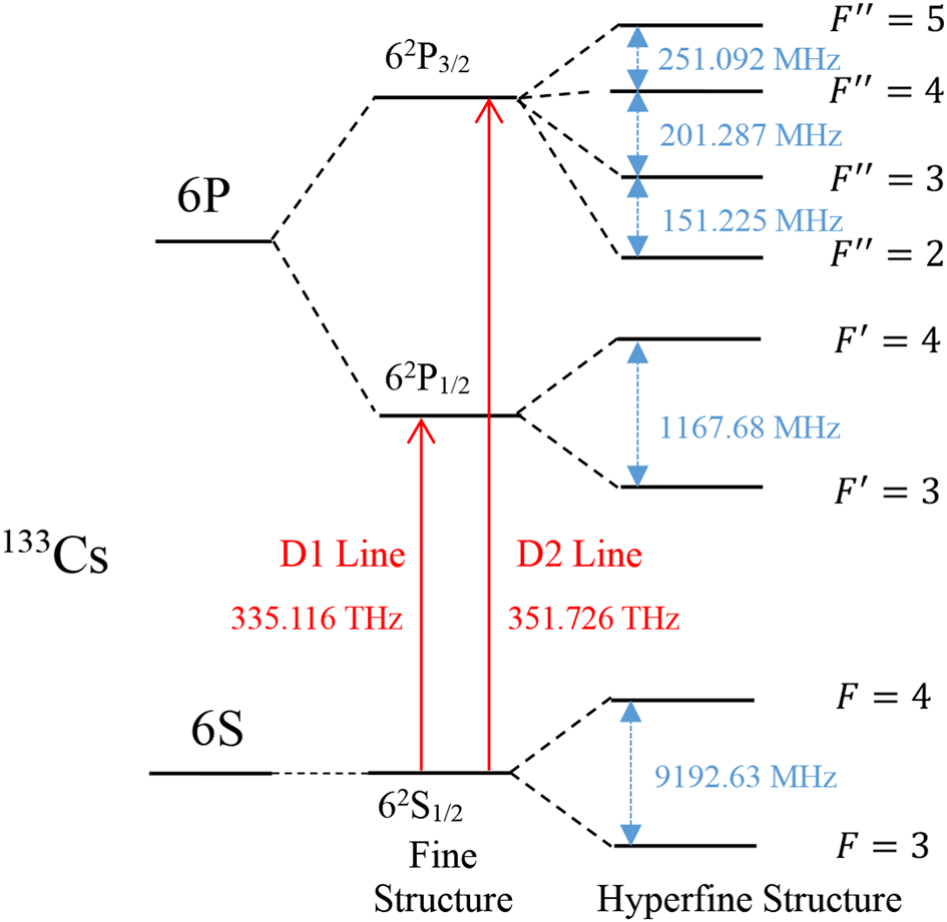
Schematic diagram of energy level splitting for a ^133^Cs atom.

Figure 1 shows the experimental setup. ^133^Cs atoms, 50 Torr of N_2_, and 100 Torr of ^4^He are filled in a cubic vapor cell (14 × 14 × 14 mm^3^) placing in a magnetic shield for preventing the interference of geomagnetic field. A magnetic field of 18.6 μT along z-axis is generated by three-axis Helmholtz coils (not shown in the figure) which also generate a rotating exciting magnetic field. ^133^Cs atoms are polarized by a left circularly polarized pump beam propagating along the z-axis. The pump light is tuned to approximately the $F = {4} \to F^{\prime}$ transition of ^133^Cs by observing the saturated absorption spectroscopy, where $F$ and $F^{\prime}$ indicate corresponding quantum numbers of the total angular momentum for the ^133^Cs atom, respectively in the 6^2^S_1/2_ and 6^2^P_1/2_ states as shown in Fig. 2. The intensity of pump light is approximately 16 μW/cm^2^, and its beam size is approximately 0.79 cm^2^. A linearly polarized probe beam propagating along x-axis monitors the x-component *P*_*x*_ of the spin polarization of ^133^Cs atoms^18,19^, and the monitoring results are extracted by a balanced polarimetry containing a polarizing beam splitter (PBS) and two photoelectric detectors (PD1 and PD2). The intensity of probe light can be controlled using a variable neutral density filter (VNFD), and its beam size is approximately 0.28 cm^2^.

## Results and discussion

**Figure 3:**
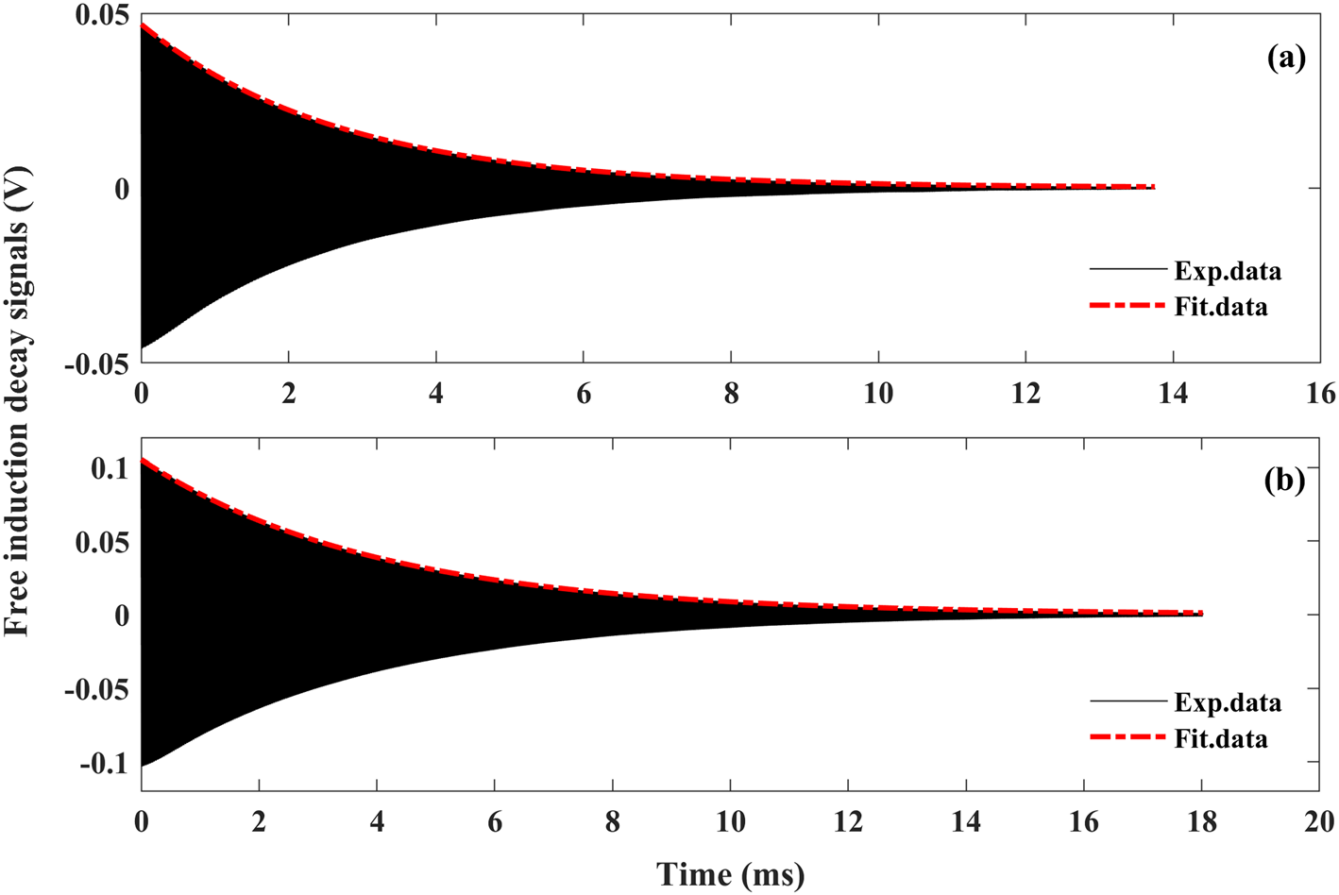
Free induction decay signals obtained by applying (**a**) clockwise rotating magnetic field and (**b**) counterclockwise rotating magnetic field.

For the ^133^Cs atoms respectively in *F* = 3 and *F* = 4 levels, since they process around the static magnetic field in opposite directions, and the numbers of their Zeeman sublevels are different, there is a large discrepancy for their transverse spin relaxation times. $T_{2 - }$ and $T_{2 + }$ are used to represent the transverse spin relaxation time for the ^133^Cs atoms in *F* = 3 and *F* = 4 levels, respectively. As the absorption rates of linearly polarized light are unequal for the alkali atoms respectively in *F* = 3 and *F* = 4 levels, the distribution of alkali atoms in the two ground-state hyperfine levels changes with the linearly polarized light, and the influences of the linearly polarized probe light on $T_{2 - }$ and $T_{2 + }$ are different. Therefore, in order to obtain an understanding of the relationship between the absorption of linearly polarized light and the transverse relaxation of ^133^Cs spin, the influences of linearly polarized light on *T*_2−_ and *T*_2+_ had better be studied separately. For realizing the above aim, $T_{2 - }$ and $T_{2 + }$ are measured under different intensities and frequencies of the linearly polarized probe light using the selectively exciting technique^20^. By selectively exciting spin-polarized ^133^Cs atoms in $F = {3}$ ($F = {4}$) level by applying a clockwise (counterclockwise) rotating magnetic field, the corresponding free induction decay signal can be obtained as shown with the black lines in Fig. 3. By fitting the free induction decay signal with the exponential function as shown with the red dashed lines in Fig. 3, *T*_2−_ and *T*_2+_ can be extracted separately^17,20^.

**Figure 4:**
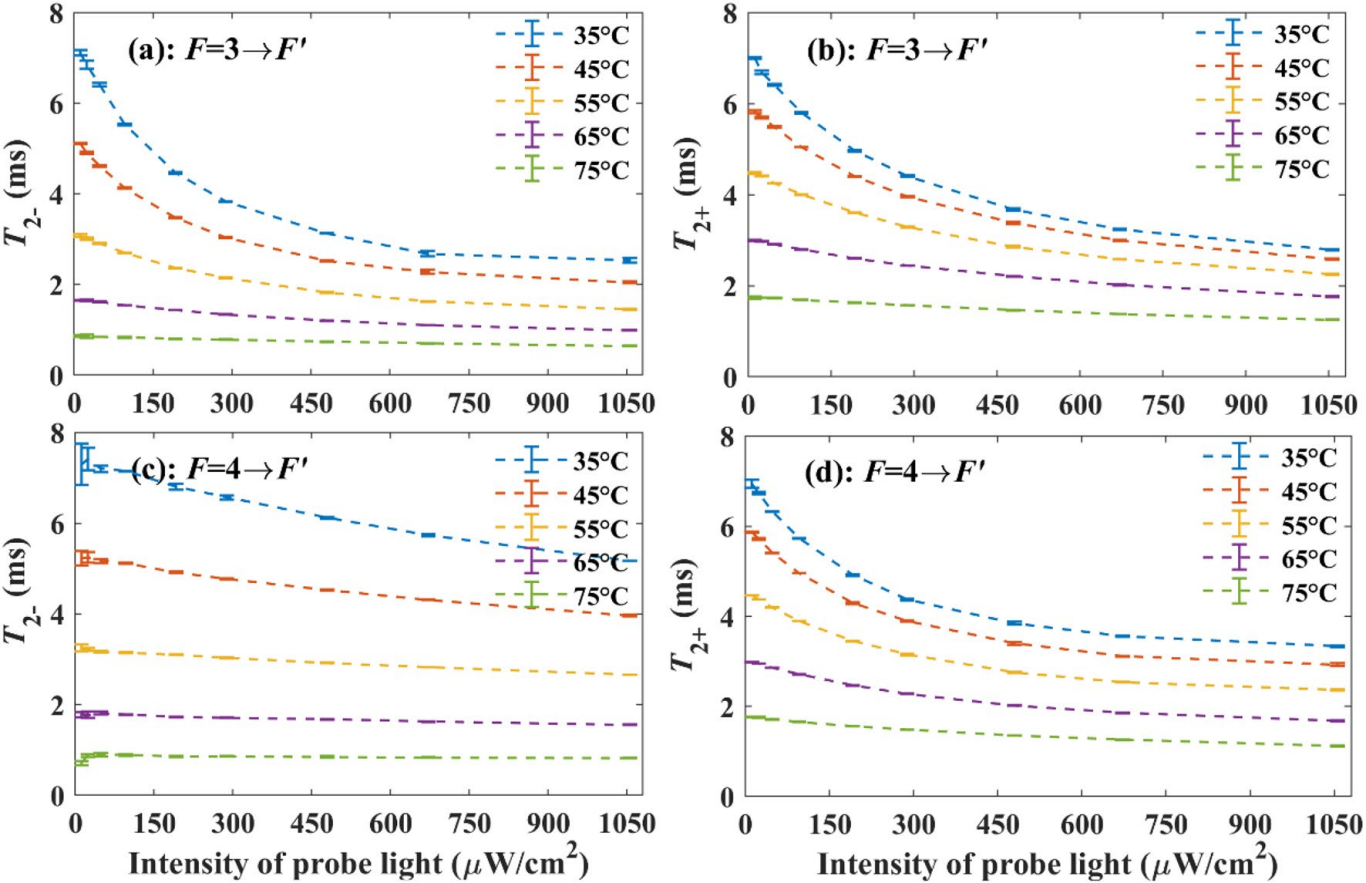
Experimentally detected $T_{2 - }$ and $T_{2 + }$ under different temperatures of the vapor cell and intensities of probe light.

For the linearly polarized probe light in practical applications, it is usually tuned to approximately a resonance frequency of D1 or D2 line of the alkali atom. Figure 4 gives the experimental results when the probe light under different intensities is tuned to approximately the $F = 3 \to F^{\prime}$ and $F = 4 \to F^{\prime}$ components of ^133^Cs D1 line, respectively, and the temperature of the vapor cell is stabilized at different values.

Since the thermal velocities and densities of ^133^Cs atoms, N_2_ and He increase with the vapor cell temperature, when the vapor cell temperature increases, the transverse spin relaxation rates caused by spin-destruction, spin-exchange and wall collisions, radiation trapping, and optical absorption grow. Therefore, we can see from Fig. 4 that $T_{2 - }$ and $T_{2 + }$ decrease when the vapor cell temperature increases. When the temperature of the vapor cell is high enough, the spin-exchange collisions will be the dominant factor causing the transverse spin relaxation for the large spin-exchange cross-section. So, as shown in Fig. 4, when the temperature is stabilized at 75 °C for the vapor cell, the influences of the intensity and frequency of probe light on $T_{2 - }$ and $T_{2 + }$ are tiny.

In order to understand the dependences of $T_{2 - }$ and $T_{2 + }$ on the intensity and frequency of probe light shown in Fig. 4, the relationship between the photon absorption rate and the probe light is discussed firstly. According to Refs.^2,10^, the absorption rates $A_{ - }$ and $A_{ + }$ of linearly polarized probe light by ^133^Cs atoms respectively in the *F* = 3 and *F* = 4 levels can be given by

1 $$A_{ - } \propto In_{ - } \sigma_{ - } , \, A_{ + } \propto In_{ + } \sigma_{ + } .$$

**Figure 5:**
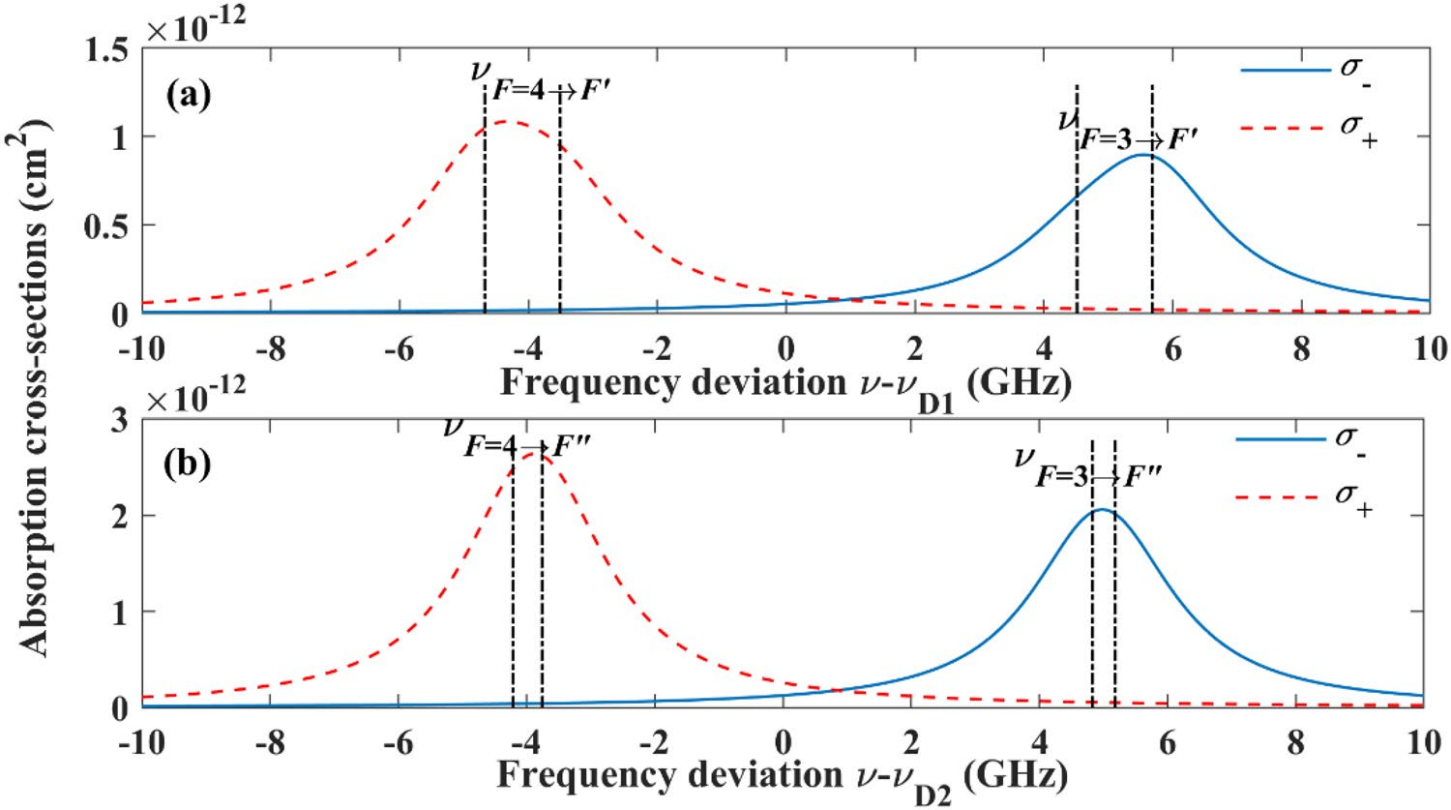
Simulating results of *σ*_−_ and *σ*_+_ for unpolarized ensemble of ^133^Cs atoms with the Lorenz broadening of 2.5 GHz when $\nu$ is surrounding (**a**) $\nu_{{{\text{D1}}}}$ and (**b**) $\nu_{{{\text{D}}2}}$.

Here, $I$ is the intensity of probe light, $n_{ - }$ and $n_{ + }$ are the densities of the ^133^Cs atoms respectively in the *F* = 3 and *F* = 4 hyperfine levels, and $\sigma_{ - }$ and $\sigma_{ + }$ are the photon absorption cross-sections for the ^133^Cs atoms, respectively in the *F* = 3 and *F* = 4 levels. In the scope of our discussion, since almost all the ^133^Cs atoms are in the ground state at any time, $n_{ + } { + }n_{ - }$ is nearly a constant. As for *σ*_−_ and *σ*_+_, they can be simulated by theoretical calculation^21^. Figure 5a,b show two simulating results for unpolarized ensemble of ^133^Cs atoms with the Lorenz broadening of 2.5 GHz, when the probe light frequency $\nu$ is surrounding the resonance frequencies $\nu_{{{\text{D1}}}}$ and $\nu_{{{\text{D}}2}}$, respectively, of the ^133^Cs D1 and D2 lines. Though under the influence of pump light and probe light, *σ*_−_ and *σ*_+_ are no more the results shown in Fig. 5, since the broadening of absorption spectrum is larger than the Zeeman splitting, the tendencies for *σ*_−_ and *σ*_+_ as a function of *ν* are similar for different distributions of ground-state ^133^Cs atoms. So, the simulating results shown in Fig. 5 can be used to estimate *σ*_−_ and *σ*_+_, approximately.

The increase of *A*_+_ (*A*_−_) means that more ^133^Cs atoms in the *F* = 4 (*F* = 3) hyperfine level will first be excited to the high levels, and then decay back to different ground-state levels quickly by quenching or spontaneous radiation, making the decoherence of atomic precession and thus leading to the transverse spin relaxation. So, $T_{2 + }$ ($T_{2 - }$) is inversely correlated with *A*_+_ (*A*_−_) in normal cases. However, if *A*_+_ (*A*_−_) is much large than *A*_−_ (*A*_+_), most ^133^Cs atoms in the *F* = 4 (*F* = 3) hyperfine level will transfer to *F* = 3 (*F* = 4) hyperfine level, causing the decrease of $n_{ + }$ ($n_{ - }$). Since the occurrence probabilities of spin-exchange collisions for the ^133^Cs atoms in the *F* = 3 (*F* = 4) hyperfine level is positively correlated with $n_{ + }$ ($n_{ - }$) at a certain operating temperature^17^, $T_{2 - }$ ($T_{2 + }$) may increase with *A*_−_ (*A*_+_) when the spin-exchange collisions is the dominant transverse spin relaxation mechanisms. According to the above basic principles, we will explain the dependences of $T_{2 - }$ and $T_{2 + }$ on the intensity and frequency of probe light.

When the probe light is tuned to approximately the $F = 3 \to F^{\prime}$ component $\nu_{{F = 3 \to F^{\prime}}}$ of ^133^Cs D1 line, with the increase of *I*, *A*_−_ will grow at first according to Eq. (1), leading to the decrease of $T_{2 - }$. However, we can find from Fig. 5a that *σ*_−_ is much larger than *σ*_+_ under this condition, so *A*_−_ will be much larger than *A*_+_, causing that $n_{ - }$ decreases with the increase of *I* at first. The decrease of *n*_−_ limits the growth rate of *A*_−_. Therefore, with the increase of *I*, $T_{2 - }$ declines quickly at first and then almost remains unchanged as shown in Fig. 4a. As for $T_{2 + }$, with the increase of *I*, $n_{ + }$ will increase at first, causing that the absorption rates of pump light and probe light by ^133^Cs atoms in the *F* = 4 hyperfine level increase with *I*, which, in turn, limits the increase of *n*_+_. Therefore, with the increase of *I*, $T_{2 + }$ declines quickly at first and then almost remains unchanged as shown in Fig. 4b.

When the probe light is tuned to approximately the $F = 4 \to F^{\prime}$ component $\nu_{{F = 4 \to F^{\prime}}}$ of ^133^Cs D1 line, with the increase of *I*,* A*_+_ will grow at first according to Eq. (1), leading to the decrease of $T_{2 + }$. However, since $\sigma_{ + }$ is much larger than $\sigma_{ - }$ under this condition, *A*_+_ will be much larger than $A_{ - }$, in addition to that the pump beam is tuned to approximately the $F = 4 \to F^{\prime}$ component of ^133^Cs D1 line, causing that $n_{ + }$ decreases with the growth of *I* notably at first. The decrease of $n_{ + }$ limits the growth rates of *A*_+_ and the absorption rate of pump light. As a result, with the increase of *I*, $T_{{2{ + }}}$ declines quickly at first and then almost remains unchanged as shown in Fig. 4d. As for $T_{2 - }$, since $n_{ - }$ increases with *I* significantly at first, causing a remarkable decrease of occurrence probability of spin-exchange collisions for the ^133^Cs atoms in the $F = {3}$ hyperfine level^17^. So, as shown in Fig. 4c, When *I* increases, the decrease rate of $T_{2 - }$ is relatively small compared with the condition when the probe light is tuned to approximately $\nu_{{F = 3 \to F^{\prime}}}$. Notably, when the temperature of the vapor cell is high enough, such as 75 °C, an unusual phenomenon that $T_{2 - }$ increases with *I* at first can be observed, since under this condition, the transverse spin relaxation caused by spin-exchange collisions is remarkable and decreases with the increase of *I* significantly.

**Figure 6:**
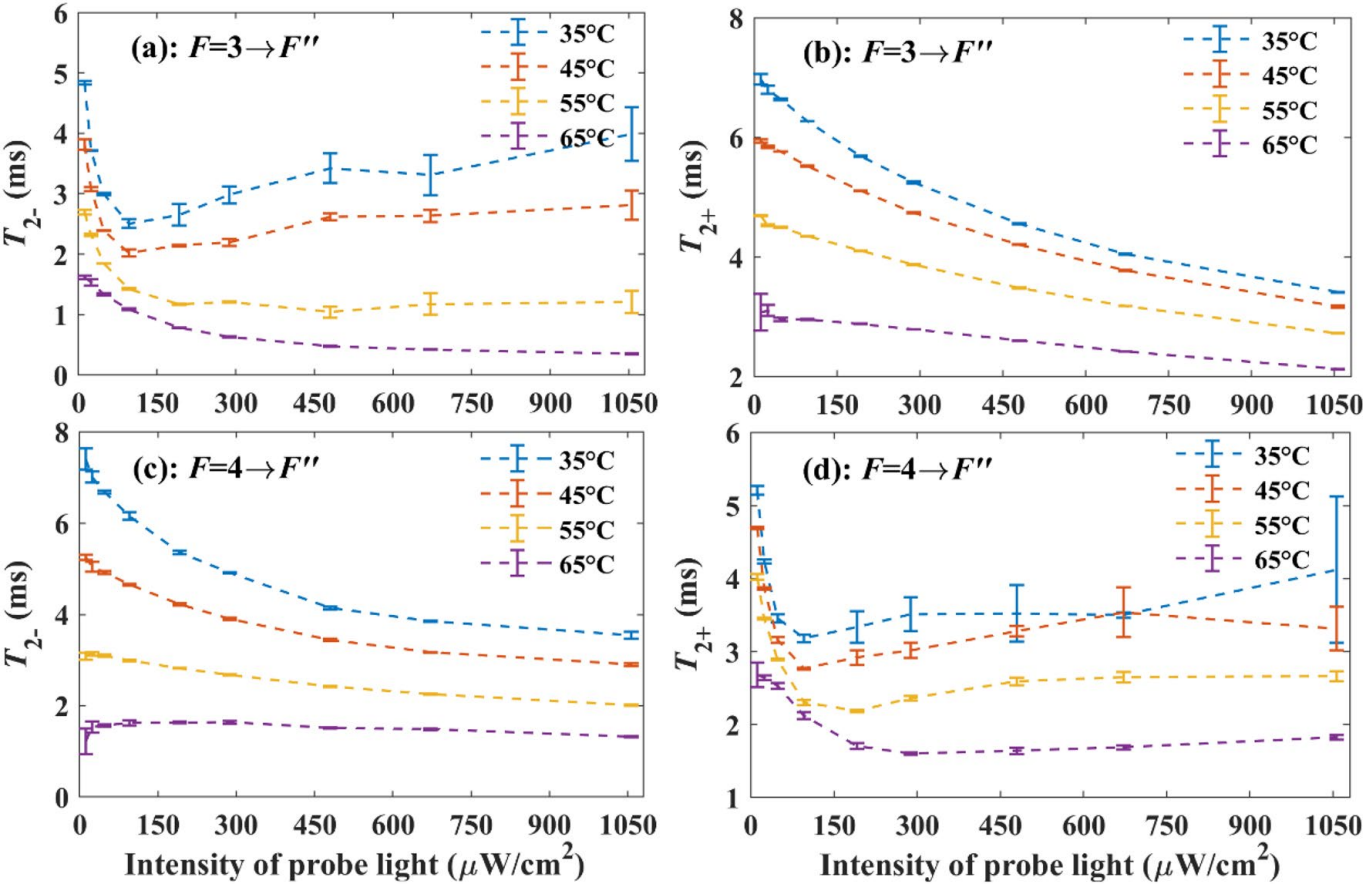
Experimentally detected $T_{2 - }$ and $T_{2 + }$ under different temperatures of the vapor cell and intensities of probe light.

In order to further observe the influence of linearly polarized light tuned to ^133^Cs D2 line on the transverse spin relaxation of ^133^Cs atoms, the probe light is tuned to approximately the $F = 3 \to F^{\prime\prime}$ and $F = 4 \to F^{\prime\prime}$ components $\nu_{{F = 3 \to F^{\prime\prime}}}$ and $\nu_{{F = 4 \to F^{\prime\prime}}}$ of ^133^Cs D2 line, respectively, and the vapor cell temperature is stabilized at different values, the experimental results under different intensities of probe light are shown in Fig. 6, where $F^{\prime\prime}$ represents the quantum number of the total atomic angular momentum for the ^133^Cs atom in the 6^2^P_3/2_ state. Compared with the condition when the probe light is tuned to approximately $\nu_{{F = 3 \to F^{\prime}}}$ or $\nu_{{F = 4 \to F^{\prime}}}$, we can observe from Fig. 5 that *σ*_−_ and *σ*_+_ are relatively large when the probe light is tuned to approximately $\nu_{{F = 3 \to F^{\prime\prime}}}$ or $\nu_{{F = 4 \to F^{\prime\prime}}}$, leading to much larger *A*_−_ and *A*_+_. As a result, when the probe light is tuned to approximately $\nu_{{F = 3 \to F^{\prime\prime}}}$ or $\nu_{{F = 4 \to F^{\prime\prime}}}$ and its intensity is small, in addition to that the vapor cell temperature is high, most probe light is absorbed by the ^133^Cs atoms, causing that the output differential signal is too weak to extract accurate $T_{2 - }$ and $T_{2 + }$. So, the results cannot be obtained precisely and thus are not shown in Fig. 6 when the temperature is stabilized at 75 °C for the vapor cell.

Compared with the condition when the probe light is tuned to approximately $\nu_{{F = 3 \to F^{\prime}}}$, when the probe light is tuned to approximately $\nu_{{F = 3 \to F^{\prime\prime}}}$, with the increase of *I*, the growth rate of *A*_−_ is much larger at first due to the relatively large *σ*_−_. Because of this, $T_{2 - }$ decreases dramatically with the increase of *I* at first as shown in Fig. 6a. Since *σ*_−_ is much large than *σ*_+_ when the probe light is tuned to approximately $\nu_{{F = 3 \to F^{\prime\prime}}}$, most ^133^Cs atoms in the *F* = 3 hyperfine level will transfer to *F* = 4 hyperfine level, causing the significant decrease of *n*_−_ and increase of *n*_+_. Especially when the temperature of the vapor cell is low, the density (*n*_−_ + $n_{ + }$) of ^133^Cs atoms is relatively small, *n*_−_ will tend to be approximately 0 due to the strong absorption of ^133^Cs atoms in the *F* = 3 hyperfine level, and *In*_−_ may decrease with *I* when *I* is large enough. So, with the increase of *I*, *A*_−_ will grow first and then decrease. When *A*_−_ decreases, the spin relaxation due to optical absorption will reduce. As a result, an unusual phenomenon that $T_{2 - }$ increases with *I* can be observed when the temperature of the vapor cell is low. When the temperature of the vapor cell is high enough, like at 65 °C, the spin relaxation due to spin-exchange collisions become significant. Since the occurrence probabilities of spin-exchange collisions for the ^133^Cs atoms in the *F* = 3 hyperfine level is positively correlated with *n*_+_ at a certain operating temperature, when *I* increases, with the significant decrease of *n*_−_ and increase of *n*_+_, though *A*_−_ will decrease with *I*, the occurrence probabilities of spin-exchange collisions for the ^133^Cs atoms in the *F* = 3 hyperfine level will always rise, causing that $T_{2 - }$ decreases with the increase of *I* all along. As for $T_{{2{ + }}}$, since *σ*_+_ is rather small under this condition, *n*_+_ will increase with *I* gradually, causing that $T_{{2{ + }}}$ declines with the increase of *I* gradually as shown in Fig. 6b.

Compared with the condition when the probe light is tuned to approximately $\nu_{{F = 4 \to F^{\prime}}}$, when the probe light is tuned to approximately $\nu_{{F = 4 \to F^{\prime\prime}}}$, with the increase of *I*, the growth rate of *A*_+_ is much larger at first due to the relatively large *σ*_+_. In addition to that the pump light is tuned to approximately the $F = 4 \to F^{\prime}$ component of ^133^Cs D1 line, causing that *n*_+_ and $T_{{2{ + }}}$ decreases with the increase of *I* notably at first. Due to the similar reason as presented in the above paragraph, an unusual phenomenon that $T_{{2{ + }}}$ increases with *I* can be observed when the temperature of the vapor cell is low as shown in Fig. 6d. As for $T_{2 - }$, since *n*_−_ increases with *I* significantly at first, an unusual phenomenon that $T_{2 - }$ increases with *I* at first can also be observed when the vapor cell temperature is stabilized at 65 °C as shown in Fig. 6c. Since compared with the condition when the probe light is tuned to approximately $\nu_{{F = 4 \to F^{\prime}}}$, *σ*_−_ is much larger when the probe light is tuned to approximately $\nu_{{F = 4 \to F^{\prime\prime}}}$, the decrease rate of $T_{2 - }$ is relatively large, leading to the different tendencies of $T_{2 - }$ shown in Figs. 4c and 6c.

## Conclusions

In summary, we have investigated the influence of linearly polarized light on the transverse spin relaxation of ^133^Cs atoms in different ground-state hyperfine levels. The situations when the linearly polarized light is adjusted to approximately a resonance frequency, respectively, of ^133^Cs D1 line and D2 line are considered, and the transverse spin relaxation times are separately detected for ^133^Cs atoms in the two ground-state hyperfine levels under different frequencies and intensities of the linearly polarized light and temperatures of the vapor cell using the selectively exciting technique. The experimental results under different conditions are compared and analyzed theoretically based on the simulation results of photon absorption cross-sections and the statistical model shown in reference^17^. Notably, the transverse relaxation time increasing with the intensity of the linearly polarized light is observed under different conditions, which is unusual for common experiments, but also well explained theoretically in this paper.

The experimental results show that through influencing the rates of optical absorption and spin-exchange collisions, the linearly polarized light plays a remarkable role in the transverse spin relaxation of alkali atoms in different ground-state hyperfine levels, which should be taken seriously in practical applications for finding the optimal intensity and frequency of linearly polarized light.

## Data Availability

The datasets used and/or analysed during the current study available from the corresponding author on reasonable request.
